# Subliminal mortality salience does not increase physical strength output in double-blind randomized controlled trial

**DOI:** 10.3389/fpsyg.2023.1321552

**Published:** 2023-12-19

**Authors:** Christopher T. J. Bartenschlager, Petra Jansen

**Affiliations:** Faculty of Human Sciences, University of Regensburg, Regensburg, Germany

**Keywords:** terror management theory, subliminal mortality salience, mindfulness, self-esteem instability, physical strength

## Abstract

**Introduction:**

Using the morality salience paradigm, this research tested whether subliminal death stimuli lead to increased physical strength. Moreover, it was investigated if mindfulness and self-esteem instability influence terror management.

**Methods:**

In total, data from 160 undergraduate sports students were analyzed. Participants completed a word decision task in which they were presented with either the word death or pain for 28.5 ms. Before and after the task, their grip strength was measured using a hand dynamometer.

**Results:**

Linear mixed models could neither confirm the effect of the mortality salience hypothesis on strength nor an influence of mindfulness and self-esteem.

**Discussion:**

The results raise the question of a potential influence of subliminal mortality salience on athletic performance and how mindfulness and self-esteem instability affect terror management.

## 1 Introduction

Humans differ in their reaction to death. Terror Management Theory (TMT; Greenberg et al., 1986) addresses this impact from a social psychological perspective and has been applied in various areas, like the investigation of physical and psychological health and the relation of TMT to close relationships, religion, and politics (Arrowood and Cox, 2020). Here, it will be investigated under which condition the reactions to death are related to a specific motor performance. TMT assumes that death awareness fuels a potentially ever-present danger that must be handled (Greenberg et al., 1986, 1990). According to TMT, this is done through cultural belief systems, the associated worldviews and self-esteem. Simply put, self-esteem and cultural worldviews have the role of an anxiety buffer. However, individual differences in self-esteem (Harmon-Jones et al., 1997) and mindfulness (Niemiec et al., 2010) shape our responses to thoughts of mortality. One objective of this study is to investigate these constructs following mortality salience (MS), which is the experimental confrontation with death-related thoughts (Burke et al., 2010).

### 1.1 Subliminal mortality salience manipulation

The Dual Process Model of TMT was introduced by Pyszczynski et al. (1999) and described the processes occurring when confronted with death-related stimuli. Following that, distal defenses emerge after MS manipulation, either after a delay or immediately, and when death-related thoughts are at the threshold of consciousness, i.e., highly available but still unconscious (Cox et al., 2019). Distal defenses involve upholding one’s worldview and pursuit of self-esteem, leading to the decline of death-thought accessibility, and an anxiety-buffering effect emerges (Greenberg et al., 1990). In approximately 80% of studies, explicit (supraliminal) death primes are used, which typically consist of answers from participants to questions regarding their emotions concerning their own death (Burke et al., 2010). By contrast, subliminal death primes were investigated less frequently and could have higher ecological validity. For example, Arndt et al. (2001) presented the word *death* under the perception threshold for 28.5 ms in a word-relation task. This led to a more negative evaluation of an author who had written an anti-U.S. essay among Americans. Regarding the underlying mechanisms of the model, there is convincing evidence that subliminal primes lead to increased death-thought accessibility (Hayes et al., 2010; Steinman and Updegraff, 2015). In summary, the effects of subliminal MS are comparable to typically used explicit methods, albeit with some advantages like concealment of manipulation and better stimulus control. Since most studies have used the more easily implementable explicit manipulation, the current study uses a subliminal one.

### 1.2 Mindfulness and terror management

Mindfulness can be seen as the ability to be present in the moment in a non-judgmental way (Kabat-Zinn, 1994). According to Bishop et al. (2004), mindfulness is a construct consisting of two components: The first involves self-regulation of attention to immediate mental experiences. The second is a specific orientation characterized by curiosity, acceptance, and openness to the present moment. A basic assumption is that mindful individuals engage intensively with death-related thoughts after a MS, and therefore no need for defense mechanisms emerges. This hypothesis was supported by Niemiec et al. (2010), who demonstrated fewer defensive reactions in more mindful individuals. Since then, few studies have been published investigating mindfulness following MS. In studies by Kashdan et al. (2011) and Grevenstein and Bluemke (2016), neither the typical MS effect nor an interaction effect was observed. However, both studies probably implemented a too short delay between MS and the dependent variable, as only a 20-item questionnaire (Grevenstein and Bluemke, 2016) and an unspecified mood assessment (Kashdan et al., 2011) served as a filler. Park and Pyszczynski (2017) found evidence consistent with Niemiec et al. (2010) in three studies. Both quasi-experimental procedures and fully randomized experiments showed no worldview defense following MS in meditating individuals and meditating and non-meditating Buddhists. This is important due to positive associations between meditation experience and scores on trait mindfulness (Lykins and Baer, 2009). Lastly, Chittaro et al. (2017) addressed facets of mindfulness and MS. For the subscales of the Five Facet Mindfulness Questionnaire (Baer et al., 2006), a marginally significant interaction between MS and acting with awareness emerged. So far, the findings indicate that mindfulness could make defensiveness redundant, or that mindful individuals process mortality-related stimuli non-reactively. This is in line with findings linking mindfulness with higher emotion regulation and lower emotional reactivity. In this sense, Heppner et al. (2015) argue that decentering mitigates reactions to self-threatening stimuli. Mindfulness is also associated with self-regulation, which is associated with less automatism (Brown and Ryan, 2003).

### 1.3 Self-esteem and terror management

Like mindfulness, studies have shown that self-esteem, which is an overall assessment of the value of one’s self, influences the effect of MS on defenses. Harmon-Jones et al. (1997) demonstrated that induced and dispositional high self-esteem was associated with an absence of worldview defense. Moreover, low implicit combined with high explicit self-esteem were found to produce high defensiveness (Schmeichel et al., 2009). Of particular interest is the research of Peters et al. (2005), who showed that MS increased physical strength of the hand. Though, this applied to individuals for whom weightlifting was perceived as important and therefore derive self-esteem from this domain. This effect was not observed for individuals who had indicated that lifting weights was not important. This is consistent with TMT, since following MS subjects strive to meet the standards on which their self-worth is based (Pyszczynski et al., 2015). Interestingly, this effect was also seen in individuals who do not generate self-esteem from strength and has been investigated by Kawakami et al. (2018). They implemented a co-activation of death-related and self-related thoughts for subjects with low muscular self-esteem. However, low muscular self-esteem individuals receiving self- and death-related words showed higher muscle strength at post-measurement than before.

Moreover, improved performance of basketball players following MS underpins the role of task-related self-esteem (Zestcott et al., 2016). Further, following MS, individuals with high body-related self-esteem have been shown to identify more with their bodies and display greater appeal in sex (Goldenberg et al., 2000) and will express environmental concern if their self-esteem is built on it (Vess and Arndt, 2008).

Even self-esteem is central for terror management, specific facets like implicit self-esteem were not investigated yet. Because research has shown that measures of implicit self-esteem have limitations (Buhrmester et al., 2011) self-esteem instability, defined as the stability or instability of self-esteem over time (Zeigler-Hill, 2006), might be a relevant construct to investigate and clarify the inconsistent results of trait self-esteem. Accordingly, a person’s self-worth can change, and a higher variability is therefore associated with a greater instability of self-esteem. For instance, the combination of high instability and high explicit self-esteem is referred to as fragile high self-esteem and “reflects positive feelings of self-worth that are vulnerable to threat, as they require continual bolstering, protection, and validation through various self-protective or self-enhancement strategies” (Kernis, 2005, p. 1590). Consequently, considering the instability component of self-esteem seems vital in the light of MS effects.

### 1.4 Goal of this study

The present study investigates the influence of the stability of self-esteem and dispositional mindfulness following exposure to subliminal MS-cues with the dependent variable grip strength. Self-esteem as well as dispositional mindfulness are chosen because both variables are assumed to lead to a less defense behavior also about death related thoughts. For the first hypothesis, we expect MS to increase handgrip force in a posttest (vs. pretest) compared to the control group. Hypothesis two expands the latter and states that lower mindfulness scores will be associated with a greater increase compared to higher ones following MS. Third it is hypothezised that high instability of self-esteem is associated with stronger handgrip force in a posttest compared to low instability. However, all hypotheses are only expected to be valid provided that strength-self-esteem is essential to the participants.

## 2 Method

### 2.1 Participants

The required sample size was calculated using G*Power (Faul et al., 2007) for a linear model. We were guided by the small to medium effects of relevant research (Peters et al., 2005; Niemiec et al., 2010; Kawakami et al., 2018). Consequently, an effect of *f*^2^ = 0.1 with α = 0.05, (1-β) = 0.8, and eight predictors (condition, time, strength self-esteem, mindfulness and self-esteem instability, interactions for three hypotheses) provided a sample size of 159 individuals. Given that linear mixed models have higher power, the number of subjects should be adequate (Hilbert et al., 2019). To account for potential drop-out in measuring self-esteem stability, we collected data from 165 participants between 19 and 32 years. Recruitment of participants was carried out through internal email newsletters and faculty homepage announcements. In appreciation for their participation, course credit was granted to the participants. All participants met the minimum age requirement of 18 years. Of the 165 participants, 163 were enrolled in the Applied Movement Science program, and three did not complete the daily life assessment, resulting in a sample of 160 participants (52.5% female, mean age = 22.3, SD age = 2.11). Inclusion criteria were the ability to exert grip strength (no injuries) and owning a smartphone. No exclusion criteria were applied. The Ethics Committee of the University of Regensburg approved the study (22-2840-101).

### 2.2 Materials and procedure

Participants were randomly assigned to one of two conditions. Embedded in a simple word decision task on the computer, they were subliminally presented with either the German word for death (Tod) or pain (Schmerz). The words were shown on a 17-inch screen with 60 Hz in black font on a light gray background using OpenSesame (Mathôt et al., 2012). The task was adopted from previous research (Arndt et al., 1997, 2001; Koole and Van den Berg, 2005). Three words were presented consecutively per trial, and the participants had to decide whether the first and third words, visible for 356 ms, were related or not (e.g., rose and tulip vs. vase and plaster). The first and third words always consisted of two syllables. The second word was presented for 28.5 ms and used for manipulation. Subjects indicated their decision by clicking the mouse buttons. There were 40 trials in total, with the second word in the first 10 trials being neutral, the next 20 trials being either death or pain, and the last 10 being senseless. For the neutral trials, words were chosen from the Berlin Affective Word List Reloaded with an emotional valence close to or equal to zero (Võ et al., 2009). Furthermore, the order of all word pairs was randomized.

Before and after the task, the maximum grip strength was measured twice using the Baseline BIMS Digital Grip Dynamometer (Fabrication Enterprises, n.d.). From both attempts the mean was calculated and used for the measurement of maximum grip strength. Subjects sat in an upright position with their elbows open at 90 degrees. The experimenter then counted from one to three, with the participants increasing the force and holding the same for one second at three (Kluttig et al., 2020). Participants have not been verbally motivated but informed about their maximum grip strength at the end. Following the central part of the study, questionnaires were assessed.

#### 2.2.1 Mindfulness

The Comprehensive Inventory of Mindfulness Experiences (CHIME; Bergomi et al., 2014) is a validated questionnaire in German Language and measures trait mindfulness with 37 items (e.g., “When I have pain, I try to avoid this perception as much as possible.”) and eight subscales: accepting nonjudgemental attitude, acting with awareness, nonreactive decentering, openness to experience, awareness of thoughts’ relativity, awareness of internal experiences, awareness of external experiences and insightful understanding. Answers were given on a 6-point scale ranging from *almost never* to *almost always*, whereas higher scores represent higher mindfulness. The internal consistency in this study was good (α = 0.86).

#### 2.2.2 Instability of self-esteem

Instability of self-esteem was measured in daily life twice on each of five consecutive days (Kernis, 2005) using the German version of the Rosenberg Self-Esteem Scale (von Collani and Herzberg, 2003), which comprises 10 items (e.g., “I have found a positive attitude toward myself.”) on a 4-point scale (0 = *strongly disagree*, 3 = *strongly agree*). The questionnaire was adapted to refer to the present moment rather than how people generally feel about themselves. Data was collected with the PIEL survey platform developed for smartphone devices (Jessup et al., 2012). Participants were prompted to complete the questionnaire at 9 a.m. and 7 p.m., for which they were given 1 h. If the questionnaire was not completed at the notification, three reminders appeared throughout the hour (after 3, 30, and 45 min). We calculated the individual standard deviation of self-esteem to obtain a measure of self-esteem instability, with higher values representing higher instability (Webster et al., 2017). A 10-point scale was used for this purpose (Kernis et al., 2008). Subjects were excluded if they completed less than 7 out of 10 assessments.

#### 2.2.3 Positive and negative affect

We used the German Version of the Positive and Negative Affect Schedule (Watson et al., 1988; Breyer and Bluemke, 2016) right before and after the central part to investigate the potential effects of the subliminal induction. The instruction aimed to provide information about the present moment. Responses were provided on a 5-point scale (1 = *not at all*, 5 = *fully*). Separate means for positive and negative effects were computed.

#### 2.2.4 Strength self-esteem

Subjects responded to three items based on the research of Peters et al. (2005) and Zestcott et al. (2016). Specifically, we asked: “How important is physical strength to you?,” “To what degree does your physical strength influence your self-esteem or how good you feel about yourself?” and “How important are strength training exercises to you?.” A 9-point scale ranged from *not at all (important)* to *fully (important)* and a higher score from the calculated mean reflects increased self-esteem, which depends on physical strength. The internal consistency was good (α = 0.83).

#### 2.2.5 Awareness check

To investigate whether the subliminal words were consciously perceived, we assessed three questions at the end of the study. Subjects were instructed to report how many words they saw in the word task per trial. If they said they saw more than two words, they had to name the word. Then they were to say which of the following five words it could have been. In randomized order, the German words for death, suffering, pain, failure, and shame were offered for choosing.

### 2.3 Design and statistical analysis

The design is 2 (condition: death vs. pain) × 2 (grip strength: pre vs. post) mixed-factorial, with the first factor being between- and the latter within-subject. Statistical analyses were performed in R (version 4.0.3; R Core Team, 2020) using lme4 package for the linear models (version 1.1.28; Bates et al., 2014). However, in a first step we analyzed repeated measures ANOVAs to investigate the dependent variables of positive and negative affect, separately. Next, we tested whether our manipulation was not consciously processed with a Pearson chi-square test, which investigates the frequencies of the five words depending on the condition. Data for the main hypotheses were analyzed with linear mixed models building on Matuschek et al. (2017) with maximum likelihood estimation and the optimx package (version 2022-4.30; Nash et al., 2022), for the exact description see supplementary material.

## 3 Results

### 3.1 Awareness check

A Pearson chi-square test revealed no different frequencies in naming the words between the conditions, $\chi^{2}$ (4) = 1.62, *p* = 0.805. Therefore, we conclude that there was no conscious awareness of the subliminal words.

### 3.2 Positive and negative affect

To test the possible effects of our manipulation on positive (PA) and negative (NA) affect, we conducted two 2 (condition: pain, death) × 2 (time: pre, post) mixed ANOVAs. For PA, a main effect of condition emerged, *F*(1, 158) = 5.23, *p* = 0.024, η^2^_p_ = 0.32. PA was significantly lower for the death condition, independently from the time factor, *F*(1, 158) = 3.02, *p* = 0.084, η^2^_p_ = 0.02. For NA, again a significant main effect of condition was observed, *F*(1, 158) = 5.76, *p* = 0.018, η^2^_p_ = 0.04. The pain condition was associated with higher NA values. Moreover, the main effect of time was significant, *F*(1, 158) = 71.16, *p* < 0.001, η^2^_p_ = 0.31. This indicates that both groups were lower on NA after the manipulation. There were no significant interactions condition x time for PA and NA. Consequently, the manipulation did not lead to an increase in either positive or negative affect.

### 3.3 Main results

Linear mixed effects models were calculated for the dependent variable strength, which was averaged for both measurements before and after the manipulation. Descriptively, the grip strength in the death condition was *M* = 38.04 (SD = 11.25) at the first and *M* = 37.55 (SD = 11.47) at the second time point – in the pain condition, *M* = 36.75 (SD = 10.42) at the first and *M* = 36.14 (SD = 10.94) at the second time point. The predictors were entered in the mixed model: time (pre vs. post), condition (pain vs. death), strength self-esteem, self-esteem instability, mindfulness, as well as their associated interactions. In the first step, the LR-test led to removal of the by-subject random slope for time, $\chi^{2}$ (2) = 2.41, *p* = 0.299, resulting in an intercept only model. This final model was used for the evaluation of significant fixed effects of interest (Table 1). The next model tested the four-way interaction of mindfulness × time × condition × strength self-esteem, which investigates the hypothesis that less mindful individuals show a strength increase following MS. However, the result of the LR-test was not significant, $\chi^{2}$ (1) = 0.18, *p* = 0.673. Next, we compared the basis model with one without the four-way interaction of self-esteem instability x time x condition x strength self-esteem, which examines whether a higher self-esteem instability leads to a higher grip strength after MS. Again, the LR-test showed no significant result, $\chi^{2}$ (1) = 0.51, *p* = 0.477. Lastly, a model comparison without the three-way interaction of strength self-esteem x condition x time, analyzing the profound effect from previous studies, also resulted in a zero effect of the same, $\chi^{2}$ (1) = 0.151, *p* = 0.698. In summary, the hypotheses were rejected. As can be seen in Table 1, strength self-esteem, mindfulness, their interaction, as well as the interaction of mindfulness and self-esteem instability reached significance when predicting grip strength with all other fixed effects in the final model. However, these results are no preregistered object of investigation and unrelated to the MS-hypothesis. Finally, the low marginal *R*^2^ (0.368) compared to the high conditional *R*^2^ (0.971) suggests that variation in grip strength is largely explained by individual differences.

**Table 1:** Final linear mixed effect model for the dependent variable grip strength.

	Grip strength			
	Estimate	*SE*	*t*	*p*
**Fixed effects**				
Intercept	−275.90	107.52	−2.57	**0.011**
Condition	−114.61	214.75	−0.53	0.594
Time	−11.18	33.86	−0.33	0.742
SSE	46.96	15.49	3.03	**0.003**
Mindfulness	79.56	26.61	2.99	**0.003**
SEI	130.84	70.82	1.85	0.066
**2-way-interactions**				
Condition*time	27.92	67.73	0.41	0.680
Condition*SSE	32.29	30.68	1.05	0.294
SSE*time	3.40	4.94	0.69	0.491
Condition*mindfulness	−1.11	53.43	−0.02	0.983
Time*mindfulness	2.95	8.45	0.35	0.727
SSE*mindfulness	−11.60	3.81	−3.05	**0.003**
Condition*SEI	80.93	141.03	0.57	0.567
Time*SEI	0.76	21.95	0.04	0.972
SSE*SEI	−19.88	10.52	−1.89	0.060
Mindfulness*SEI	−34.88	17.70	−1.97	**0.050**
**3-way interactions**				
Condition*time*SSE	−3.84	9.87	−0.39	0.698
Condition*time*mindfulness	−7.32	16.89	−0.43	0.665
Condition*SSE*mindfulness	−2.93	7.60	−0.39	0.701
Time*SSE*mindfulness	−0.90	1.22	−0.74	0.461
Condition*time*SEI	−32.44	43.89	−0.74	0.460
Condition*SSE*SEI	−22.76	20.83	−1.09	0.275
Time*SSE*SEI	−0.84	3.28	−0.26	0.798
Conditon*mindfulness*SEI	−8.53	35.33	−0.24	0.809
Time*mindfulness*SEI	−0.39	5.50	−0.07	0.944
SSE*mindfulness*SEI	5.03	2.63	1.91	0.057
**4- and 5-way interactions**				
Condition*time*SSE*mindfulness	1.04	2.44	0.43	0.670
Condition*time*SSE*SEI	4.67	6.56	0.71	0.477
Condition*time*mindfulness*SEI	8.66	10.99	0.79	0.432
Condition*SSE*mindfulness*SEI	3.66	5.22	0.70	0.484
Time*SSE*mindfulness*SEI	0.25	0.82	0.30	0.764
Condition*time*SSE*mindfulness*SEI	−1.27	1.64	−0.77	0.440
**Random effects**				
σ^2^	7.62			
τ_00 participants_	159.45			
ICC	0.95			
Observations	320			
Marginal *R*^2^/Conditional *R*^2^	0.368/0.971			

SSE = strength self-esteem, SEI = self-esteem instability, time = pre- vs. post-manipulation, condition = death vs. pain, mindfulness = global mindfulness score. Significant results are printed in bold.

## 4 Discussion

The study aimed to investigate whether subliminal presentation of the word death influences athletic performance. Based on a TMT-framework, we hypothesized that MS leads to an increase in muscle strength compared to a control condition for participants rating their physical strength as important for their self-worth. However, the relevant interaction did not reach significance. In addition, two variables were investigated that potentially influence this effect: mindfulness and self-esteem instability. It was stated that lower mindfulness and higher self-esteem instability would be associated with higher strength following reminders of death. Again, none of the hypotheses was confirmed.

### 4.1 Theoretical implications

These results seem to contradict numerous studies that reliably demonstrated TMT effects over many years (Burke et al., 2010; Cox et al., 2019). Our results could provide insights on the boundary conditions and robustness of MS effect. They hint that the MS effect might appear only under specific conditions. Furthermore, the results align with the evidence that the impact may be smaller and not as universal as assumed (Klein et al., 2019; Schindler et al., 2021).

The question arises why other studies demonstrate effects of MS in motor behavior. Several reasons could be carved out: One difference to the study of Peters et al. (2005) was implementation of a subliminal instead of supraliminal manipulation and a different population. It is plausible that lifting weights was more important to weightlifting individuals than sports students. However, Kawakami et al. (2018) showed that an ordinary subliminal MS was insufficient to induce increased grip strength in undergraduates. Unfortunately, the authors did not measure task-related self-esteem. Lastly, the effects vary in the different cultures of the studies and Americans react stronger to MS induction than Europeans (Burke et al., 2010). A compatible explanation would be that the participants showed lower levels of death anxiety than typical samples participating in TMT studies. However, those possible cultural differences in diverse reaction to levels of death anxiety must be investigated in more depth.

Moreover, the manipulation was conducted under the threshold of conscious awareness. The presentation of the stimuli and the procedure of the masked priming were very similar to what had been used in previous studies (e.g., Koole and Van den Berg, 2005). One modification is the presentation of 20 trials with the word death or pain and 20 trials with distractor items. Other studies used, for example, 10 death and 30 distractor trials (Arndt et al., 2001), only 10 death trials (Arndt et al., 1997) or 25 death and 25 distractor trials (Kawakami et al., 2018). This gives no reason to believe the alteration is a factor contributing to the zero effect results and is also supported by the fact that far less standardized MS inductions have resulted in distal responses (Zestcott et al., 2016). In addition, there is evidence in a meta-analysis on masked priming that the number of trials can influence the priming effect – with a higher number being associated with larger effect sizes for lexical decision and naming tasks (van den Bussche et al., 2009). To check whether the induction indeed led to increased death though accessibility, a rather complicated measurement of the same would have been necessary.

The present study is the first to investigate mindfulness and self-esteem instability following subliminal death reminders. The absence of an effect for mindfulness is essential considering the findings of Niemiec et al. (2010). To our knowledge, their research was the only one to show significant buffering effects using the Mindful Attention and Awareness Scale (MAAS; Brown and Ryan, 2003) for mindfulness. However, the MAAS has limitations: There are serious concerns as to whether the MAAS measures mindfulness (e.g., van Dam et al., 2018), in particular, it does not measure the acceptance component (Sauer et al., 2013) and does not measure non-judgmental awareness (Baer et al., 2006). Due to these issues, we used the CHIME which is based on all previous operationalizations of mindfulness (Bergomi et al., 2014). Nevertheless, the corresponding hypothesis did not reach significance. But overall, the relevant literature suggests that mindful individuals process death-related stimuli differently (e.g., Park and Pyszczynski, 2017). Though, the results encourage the investigation of different questionnaires when using TMT-paradigms.

Lastly, a possible influence of self-esteem instability was supposed due to similar results on other facets of self-esteem in TMT research (Rothschild et al., 2019). One explanation for the unconfirmed hypothesis could be that self-esteem instability has sometimes been considered with the global self-esteem level. However, without accounting for the global level of self-esteem, self-esteem instability was also associated with relevant variables like verbal defensiveness (Kernis et al., 2008). Relevant research demonstrated relations between instability and defense styles. In this sense, Zeigler-Hill et al. (2007) showed associations between self-esteem instability and immature defenses like rationalization.

### 4.2 Practical implications

The study provides evidence that it is not that easy to boost motor-performance with one short single psychological priming intervention. To evoke death anxiety through priming seemed not to be a relevant method which should applied in sport science as a short psychological method for performance improvement. If there are effects with other methods of mortality salience and with relation to the dispositional mindfulness and the self-esteem of the person, the effects seemed to be rather small. Psychological training for motor performance improvement needs time. Students should be aware that there seemed to be no psychological induced short cut for the improvement of motor performance.

### 4.3 Limitations and future research

Beside the strength of a pre-registered, well-powered study using Linear mixed-models and the fact that neither the participants nor the experimenter was aware of the condition, the study has limitations: Although the word task was designed similarly to previous studies, the one-syllable words were used for the first time as no German-language items were available from other publications. Consequently, it remains unclear whether the word pairs used could have influenced the results due to their valence or semantics. Moreover, the German words for the manipulation (Tod; Schmerz) differ more clearly in word length than the English-language originals (dead or death; pain), whereas we doubt that this can explain the absence of effects. Concerning the measurement of grip strength, it should be noted that a constant adjustment was chosen for the grip width. This was done for standardization but not the ideal setting for all participants. Lastly, the assessment of self-esteem instability in daily life faces challenges, as filling in the questionnaire takes time and can provoke reactivity. In addition, it was impossible to assess whether the subjects filled in the questionnaires accurately without being disturbed.

Finally, the work provides evidence that subliminal death confrontation does not influence individuals who do not engage in weightlifting. However, a false-negative result cannot be excluded, as TMT findings have already been replicated in many cultures and countries (Burke et al., 2010). Future studies should investigate mindfulness and self-esteem using more typically dependent variables, such as worldview defense. Finally, it should be emphasized that transparent and sufficiently powered studies, as was the case in this work, are necessary to address the question of the validity of the MS hypothesis.

## Data availability statement

The datasets presented in this study can be found in online repositories. The names of the repository/repositories and accession number(s) can be found at: https://osf.io/yh6k7/?view_only=5e29264db0c642b784a1f9b766fa7a22.

## Ethics statement

The studies involving humans were approved by Ethical committee of Regensburg University (reference number: 22-2840-101). The studies were conducted in accordance with the local legislation and institutional requirements. The participants provided their written informed consent to participate in this study.

## Author contributions

CB: Conceptualization, Data curation, Formal analysis, Investigation, Validation, Writing – original draft, Writing – review & editing. PJ: Conceptualization, Supervision, Writing – review & editing.
